# Gamma-secretase inhibitor does not induce cytotoxicity in adult T-cell leukemia cell lines despite NOTCH1 expression

**DOI:** 10.1186/s12885-022-10003-w

**Published:** 2022-10-15

**Authors:** Shinsuke Suzuki, Sawako Hourai, Kimiharu Uozumi, Yuichirou Uchida, Makoto Yoshimitsu, Hachiman Miho, Naomichi Arima, Shin-ichi Ueno, Kenji Ishitsuka

**Affiliations:** 1 Cancer Center, Kagoshima University Hospital, 8-35-1 Sakuragaoka, Kagoshima, 890-8520 Japan; 2 Department of Clinical Oncology, Course of Advanced Therapeutics, Kagoshima University Graduate School of Medical and Dental Sciences, Kagoshima, Japan; 3 Department of Hematology and Rheumatology, Kagoshima University Hospital, Kagoshima, Japan; 4 Department of Environment and Public Health, Environmental Health Section, Ministry of the Environment, National Institute for Minamata Disease, Minamata, Japan; 5 Department of Medical Oncology, National Hospital Organization Kagoshima Medical Center, Kagoshima, Japan

**Keywords:** NOTCH1, Adult T-cell leukemia/lymphoma, γ-Secretase inhibitor, Molecular pathogenesis

## Abstract

**Background:**

Activated mutations in *NOTCH1* are drivers of T-cell type acute lymphoblastic leukemia/lymphoma. The γ-secretase inhibitor (GSI), which suppresses the function of NOTCH1, is expected to be a molecular-targeted agent. NOTCH1 is also expressed in other malignant neoplasms. We aimed to determine the function of NOTCH1 expression and the effects of GSI on adult T-cell leukemia/lymphoma (ATL) caused by long-term human T-cell leukemia virus type I (HTLV-1) infection.

**Methods:**

We analyzed the expression of NOTCH1 in six ATL- and HTLV-1-infected cell lines and investigated the influence of activated NOTCH1 (i.e., the cleaved form of NOTCH1) together with GSI on cell proliferation.

**Results:**

Activated NOTCH1 found in ATL- and HTLV-1-infected cell lines was undetectable after incubation with GSI, regardless of Tax expression (HTLV-1-coded protein). Whole-exome sequencing revealed that activated *NOTCH1* mutations were undetectable in six ATL- and HTLV-1-infected cell lines, regardless of abundant NOTCH1 expression. Moreover, GSI did not suppress the growth of ATL cell lines.

**Conclusions:**

These findings suggested that NOTCH1 protein is constitutively activated but is likely a passenger during *NOTCH1*-mutation-negative ATL cell proliferation.

**Supplementary Information:**

The online version contains supplementary material available at 10.1186/s12885-022-10003-w.

## Background

*NOTCH1* was discovered by analyzing the DNA flanking the breakpoints of a recurrent t (7;9)(q34;q34.3) chromosomal translocation, which juxtaposes truncated NOTCH1 with T-cell receptor β, directing the overexpression of N-terminally truncated polypeptides, in < 1% T-cell acute lymphoblastic leukemia/lymphoma (T-ALL) [1]. Over 50% of human T-ALL has activated mutations that involve the extracellular heterodimerization domain (HD) and/or C-terminal glutamic acid, serine, and threonine (PEST) domain of NOTCH1 [2]. These findings have expanded the role of activated NOTCH1 in the molecular pathogenesis of T-ALL and provide justification for targeted therapies that affect NOTCH1 signaling. NOTCH1 normally undergoes proteolytic processing at the S1 site to form an extracellular N-terminal subunit (NEC) and a transmembrane subunit (NTM). NOTCH ligand binding to the epidermal growth factor-like repeat region of NEC stimulates metalloprotease cleavage at a second protease-cleavage site (S2) to create membrane-bound NTM*I monomers. These are subsequently cleaved at several sites within the HD that binds to extracellular and transmembrane subunits (TM) via the γ-secretase (GS) protease complex. This results in the release of intracellular domain 1 (ICN1), which forms a complex that stimulates effector transcription [3]. The ICN1 contains regulators of amino acid metabolism (RAM), ankyrin repeat (ANK), and transcriptional activation domains (TAD), and PEST, ANK, and TAD are critical for T-ALL induction in mice [4]. ICN1-TCR translocation and HD mutations resulted in increased ICN1 production, whereas PEST mutations resulted in increased ICN1 stability [2]. The γ-secretase inhibitor (GSI) might be effective against T-ALL subsets with this overactive NOTCH signaling [2].

Adult T-cell leukemia/lymphoma (ATL) is an aggressive malignancy of mature peripheral T lymphocytes that is acquired after long-term infection with human T-cell leukemia virus type I (HTLV-1). The regulatory protein Tax encoded by HTLV-1 plays a central role in the early stages of ATL. Molecular targeted approaches, unlike standard cytotoxic chemotherapy treatments, should lead to the eradication of ATL [5]. Kataoka et al. found that 15% of ATL has activating mutations in NOTCH1 [6]. Unlike exclusive activated frameshift mutations, those in ATL are single-substitution mutations in the PEST domain [6]. Kagoshima University Hospital is located in an HTLV-1-endemic area. The cell lines were randomly established from patients who underwent chemotherapy treatment at our hospital [7]. The purpose of this experiment was to explore the possibility of using NOTCH1-targeted therapies to treat patients with ATL instead of those based on NOTCH1 mutations. We therefore investigated NOTCH1 protein expression and the effects of GSI in ATL cell lines expressing NOTCH1 to determine the function of NOTCH1 in ATL.

## Methods

### Cell lines

The human ATL cell lines, S1T and Su9T01, and the HTLV-1-infected T-cell lines, Oh13T, K3T, F6T, and MT-2, were maintained in Gibco RPMI 1640 supplemented with 1% penicillin/streptomycin and 10% fetal bovine serum (all from Thermo Fisher Scientific Inc., Waltham, MA, USA). All cell lines, except MT-2, were established from patients in our laboratory [7]. We purchased MT-2 cells from the Japanese Cancer Research Resources Bank (JCRB1210; Osaka, Japan). We analyzed Tax protein-positive and -negative cell lines to determine the influence of Tax on NOTCH1 protein expression and function, as well as on cell growth. We also examined NOTCH1 protein expression in clones K3T and F6T that produce Tax, and the S1T and Su9T01 clones that do not. We also examined the T-ALL cell lines and JURKAT cells that are without a NOTCH1 mutation. JURKAT cells do have a FBXW7 mutation, which causes higher NOTCH signaling. Moreover, they have a PTEN deletion, resulting in PI3K pathway activation and thus a rescue from GSI [8, 9]. SUP-T1, with t(7;9)(q34;q34.3), resulting in aberrant expression of the NTM and absence of full-length NOTCH1, and HD-Mar, with t (9;14)(q34.3;q11.2), carrying breakpoints located inside HD, resulting in high GSI sensitivity despite NOTCH1 overexpression [10], were also examined. We used the human Burkitt lymphoma cell line Namalwa and peripheral blood lymphocytes (PBLs) from healthy individuals with or without activation. The SUP-T1, HD-Mar, and Namalwa cell lines were obtained from DSMZ (Department of Human and Animal Cell Cultures, Braunschweig, Germany). Activated PBLs were incubated with 10 U/mL of recombinant human IL-2 (Amgen Biologicals, Thousand Oaks, CA, USA) for 6 days at 37 °C in 95% humidity under a 5% CO_2_ atmosphere. S1TcTax consisted of cell lines stably transfected with the Tax expression vector pcTax WT that harbors wild-type tax cDNA and the neomycin-resistance gene. Control S1TcNeo harbored a plasmid containing the neomycin resistance gene. S1TcTax05 and S1TcTax10 clones express abundant *Tax* mRNA [7]. The Ethics Committee and Institutional Review Board of Kagoshima University approved the study, in which healthy persons provided written informed consent to participate.

### Protein analyses

Proteins were analyzed by western blotting as follows. Cells (4 × 10^6^) were lysed with 50 μL of RIPA buffer comprising 50 μL 2× SDS buffer, 1 μL aprotinin (1 mg/mL), and 5 μL of phenylmethylsulfonyl fluoride (20 ng/mL). Lysates (20 μL) were loaded onto 6% SDS-PAGE gels (Bio-Rad Laboratories Inc., Hercules, CA, USA) and blotted onto nitrocellulose membranes (Schleicher Schuell, Dassel, Germany) using a semi-dry technique. Loading was checked using Ponceau dye and an anti-human β-actin antibody (Ab) (Santa Cruz Biotechnology Inc., Dallas, TX, USA). We obtained the following NOTCH1 Abs: mN1A Ab against ANK domain (BD Biosciences, San Diego, CA, USA) and Ab8925 against TM/RAM domain in ICN1 (Abcam, Cambridge, UK). The Ab8925 epitopes require prior exposure to GS and detected GS-cleaved NOTCH1, according to the manufacturer. For accuracy, we performed western blotting analysis three times.

### Reagents

N-[N-(3,5-difluorophenyl)-L-alanyl]-S-phenyl-glycine t-butyl ester (DAPT; Peptides International Inc., Louisville, KY, USA) was dissolved in 10 mM dimethyl sulfoxide (DMSO) stock solution.

### MTT reduction assays

Cell lines were cultured for 96 h with the indicated concentrations of DAPT or DMSO. Cell viability was then determined using MTT assays (Sigma-Aldrich Corp., St Louis, MO, USA).

### Sequencing

We created libraries using Ion AmpliSeq™ Exome technology (Thermo Fisher Scientific Inc.) and then genotyped amplicons by whole-exome sequencing (WES) using an Ion Proton™ platform (Life Technologies Corp., Carlsbad, CA, USA) as described by the manufacturer. Sequences were aligned against a reference genome (GRCh37/hg19) using TMAP (Thermo Fisher Scientific Inc.), and then genotyped variants were confirmed by Sanger sequencing.

### Statistical analysis

Data are shown as means ± standard error (SE). Statistical significance was determined using Student t-tests. Values with *p* < 0.05 were considered statistically significant.

## Results

Expression of NOTCH1 protein is excessive in ATL and HTLV-1-infected T-cell lines.

**Fig. 1:**
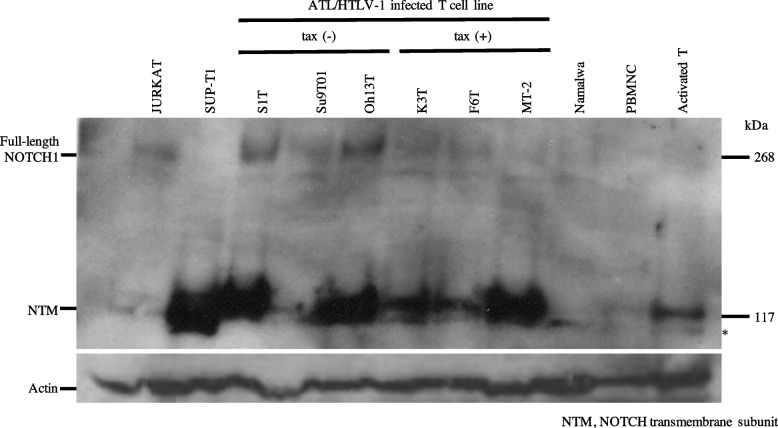
NOTCH1 protein expression of ATL and HTLV-1-infected T-cell lines. Western blots of NOTCH1-ANK domain antibody (Ab) to mN1A in ATL and HTLV-1-infected T-cell lines. Normal protein expression of full-length NOTCH1 (~ 300 kDa) and NTM (~ 120 kDa) was detected in *NOTCH1* non-translocated JURKAT cells. High protein expression of NTM (~ 120 kDa) was found in ATL and HTLV-1-infected T-cell lines, regardless of Tax expression, except Su9T01. Absence of full-length NOTCH1 bands and aberrant protein expression undercut 117 kDa wild-type polypeptide, implying *NOTCH1* translocation-driven expression in SUP-T1. Red band is non-specific

Figure 1 shows western blots of the minimal transforming regions and ankyrin domains in ATL and HTLV-1-infected T-cell lines. The JURKAT, T-ALL cell line without a NOTCH1 mutation but with a FBXW7 mutation, expressed moderate amounts of full-length NOTCH1 (~ 300 kDa) and NTM (~ 120 kDa) proteins. The ~ 300 kDa band was absent, and aberrant protein expression undercuts the 117 kDa wild-type polypeptide in SUP-T1 and NOTCH1 translocation cell lines, indicating translocation-driven expression (Fig. 1). Except for the Su9T01 cell line, all ATL and HTLV-1-infected cell lines expressed abundant NTM protein and moderate amounts of full-length NOTCH1 protein. In contrast to SUP-T1, aberrant protein expression undercuts the 117 kDa wild-type polypeptide that was undetected in all ATL and HTLV-1-infected T-cell lines. Neither full-length NOTCH1 nor NTM was detected in Namalwa, a human Burkitt B cell lymphoma cell line. Faint NTM was detected in peripheral blood mononuclear cells. In contrast, activated T-cells expressed abundant normal-sized NTM, indicating that NOTCH1 alleles are expressed not only in tumors, but also in activated normal cells (Fig. 1).

HTLV-1-Tax protein did not affect NOTCH1 protein expression in ATL and HTLV-1- infected T-cell lines.

**Fig. 2:**
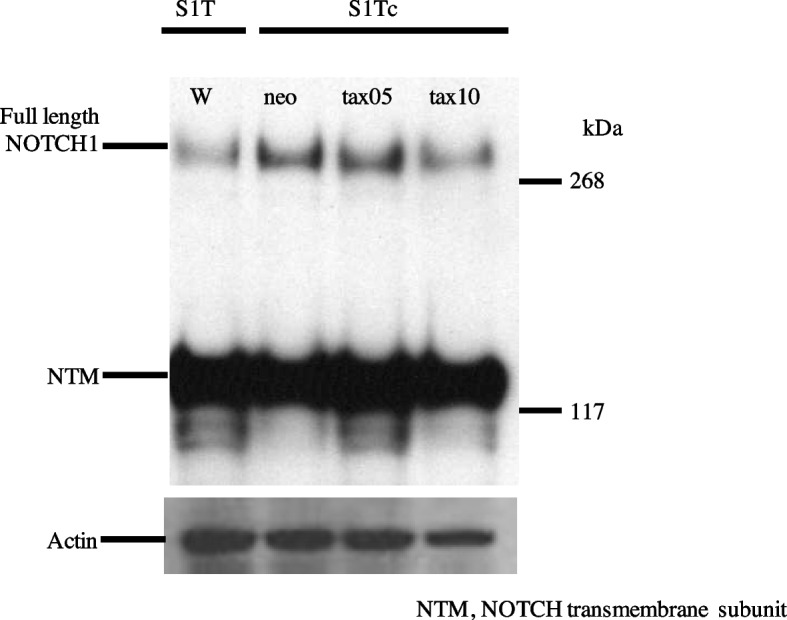
NOTCH1 protein expression in S1T cells with forced Tax expression. Western blots of mN1A in S1T cells with forced Tax expression. Control S1TcNeo cells harbor plasmids containing neomycin resistance gene. S1TcTax05 and S1TcTax10 clones expressed abundant *Tax* mRNA

We analyzed NOTCH1 protein expression in the Tax-negative S1T-cell line and a clone expressing *Tax* mRNA, S1TcTax05, and S1TcTax10 [7]. The expression of full-length NOTCH1 and NTM did not significantly differ among S1TcNeo, S1TcTax05, and S1TcTax10 (Fig. 2).

Activated NOTCH1 dissipation was affected by GSI.

**Fig. 3:**
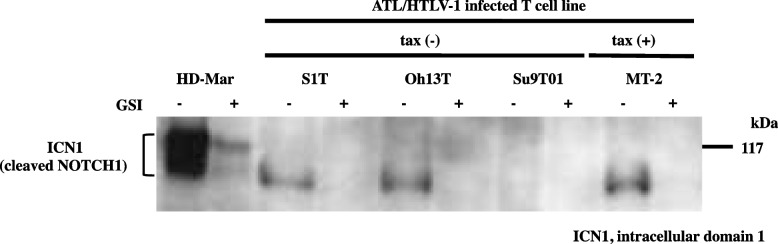
Active form of NOTCH1 following GSI treatment. NOTCH1 polypeptides were immunoprecipitated from whole-cell extracts using antibodies against GS-cleaved active NOTCH1 (ab8925). TM/RAM species (ICN1) were lost after incubation with GSI. Brackets show aberrant accumulation of TM/RAM species (ICN1) of HD-Mar with *NOTCH1* rearrangement

Activated NOTCH1 (ICN1) generated via GS-cleavage and labeled using a TM/RAM domain antibody was detected in ATL- and HTLV-1-infected T-cell lines, except for the Su9T01 cell line (Fig. 3). Activated NOTCH1 expression was more abundant in the T-ALL cell line HD-Mar with the *NOTCH1* rearrangement t (9;14)(q34.3;q11.2) [10] than in ATL and HTLV-1-infected T-cell lines without *NOTCH1* mutation (Table 1). Activated *NOTCH1* was undetectable in cells incubated with GSI, regardless of Tax expression. These findings emphasized the dependence of TM/RAM expression on GSI activity in T-ALL, HD-Mar, ATL, and HTLV-1-infected T-cell lines.

**Table 1:** NOTCH1 mutation and protein expression of ATL and HTLV-1 infected T cell lines

Cell line	Tax protein expression	NOTCH1		FBXW7 mutation
		mutation	protein expression	
**S1T**	**–**	**–**	**+**	**–**
**SU9T01**	**–**	**R879Q**	**–**	**–**
**Oh13T**	**–**	**–**	**+**	**–**
**K3T**	**+**	**–**	**+**	**–**
**F6T**	**+**	**–**	**+**	**–**
**MT-2**	**+**	**–**	**+**	**–**

Growth of ATL and HTLV-1-infected T-cell lines was not suppressed by GSI.

**Fig. 4:**
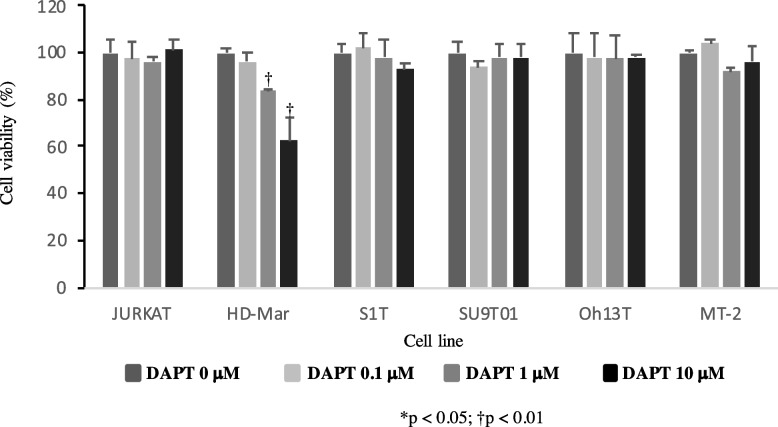
Effects of progression in ATL and HTLV-1-infected T-cell lines after incubation with GSI. T-ALL, ATL, and HTLV-1-infected T-cell lines were cultured for 96 h with indicated concentrations of DAPT or dimethyl sulfoxide (DMSO)

We assessed the effects of the potent GSI, DAPT, on the growth of ATL and HTLV-1-infected T-cell lines. After a 96-h incubation, DAPT significantly and dose-dependently suppressed the growth of HD-Mar, but not the ATL and HTLV-1-infected T-cell lines (Fig. 4). These findings suggested that *NOTCH1* was constitutively activated but probably acted as a passenger in the proliferation of ATL and HTLV-1-infected T-cell lines without *NOTCH1* mutation (Table 1).

Activated *NOTCH1* mutations were undetectable in the ATL and HTLV-1-infected cell lines.

Six ATL and HTLV-1-infected cell lines, including ATLs with single-substitution mutations in the PEST domain, were assessed by WES (DNA Data Bank of Japan repository, https://ddbj.nig.ac.jp/resource/biosample/SAMD00453566). Activated *NOTCH1* mutations, especially in the PEST domain and inactivated FBXW7 mutations, were undetectable in six ATL- and HTLV-1-infected cell lines (Table 1). However, NOTCH1 p.Arg879Gln was detected in the Su9T01 cell line with low NOTCH1 protein expression (Fig. 1).

## Discussion

GSI is useful for screening cell lines for evidence of ongoing NOTCH processing based on the accumulation of GS-cleaved NOTCH1 and its dependence on NOTCH nuclear access for growth and survival. Moderate and abundant expression of full-length NOTCH1 protein and NTM, respectively, were detected in five of six ATL, and HTLV-1-infected cell lines. Although GSI reduced the amount of GS-cleaved NOTCH1, it did not suppress the growth of these cell lines.

Unlike SUP-T1 translocated cells with aberrant NTM expression, the absence of full-length NOTCH1 implied translocation-driven expression [8]. We did not detect aberrant NOTCH1 expression in any of the ATL- and HTLV-1-infected cell lines studied (Fig. 1). As a driver oncogene mutation, translocation has a more prominent effect than gene amplification on carcinogenesis. To date, *NOTCH1* translocation in ATL has not been reported. Progression might be less dependent on the NOTCH1 signaling pathway in ATL than in T-ALL.

We found that forced expression of the Tax protein did not affect full-length NOTCH1 or NTM expression in S1T cell line without Tax expression (Fig. 2). Although Tax plays a central role in the early stages of ATL pathogenesis, we could not detect the direct role of HTLV-1 in NOTCH signaling. Activated PBLs also showed expression of NOTCH1 protein (Fig. 1). We considered that the activation of T-cells with HTLV-1 infection resulted in NOTCH1 expression. Cheng et al. reported that Tax activated NOTCH1 signaling by prolonging the half-life of NICD [11].

Pancewicz et el. reported that 30% of ATLs have PEST domain single-substitution mutations and showed that the GSI inhibition of NOTCH1 signaling reduces tumor cell proliferation and tumor formation in mice engrafted with ATL. They concluded that NOTCH1 signaling could result in cellular proliferation in ATL [12]. Although GSI affected the dissipation of the GS-cleaved NOTCH1 (Fig. 3), it did not suppress ATL- and HTLV-1-infected T-cell lines (Fig. 4). The reasons for this discrepancy at the point of NOTCH1 signaling dependence on ATL proliferation are as follows. We did not find any mutations that activated the PEST domain in any of the cell lines used in this study (Table 1). We previously described a *NOTCH1* rearrangement, t (9;14)(q34.3;q11.2), in HD-MAR and HT-1 T-ALL cell lines, both of which are dependent on the NOTCH1 signaling pathway for proliferation. GSI reduced proliferation in t (9;14)-harboring HD-MAR and HT-1 cells, where NOTCH1 is truncated inside the HD under transcriptional control of the T-cell receptor alpha variable [10]. The weaker TM/RAM epitope expression of the three ATL- and HTLV-1-infected T-cell lines than that of HD-Mar before incubation with GSI (Fig. 3) suggests a protein structural basis, and NOTCH1 signaling is much more important for the progression of T-ALL with t (9;14) than ATL without a PEST domain mutation. We previously reported that t (9;14) in HD-MAR, where NOTCH1 is truncated inside the HD, resulted in increased ICN1 production [10]. However, we considered that HTLV-1 in these cell lines resulted in increased ICN1 stability. In the future, an additional cell model based on the cell lines derived from the patients, but with an activating *NOTCH1* mutation, needs to be developed to provide a better view of the role of *NOTCH1* in ATL pathogenesis. Another reason for the discrepancy is that we could not discount the possibility that additional mutations that occurred during culture for several years might have resulted in NOTCH1 signaling becoming dispensable for growth in vitro. Furthermore, we examined high-concentration of DAPT (up to 10 μM) for 96 h, but it may be the case that the actual effect on the proliferation of ATL/HTLV-1-infected T-cell lines only occurs after long-term incubation.

We detected NOTCH1 p.Arg879Gln in Su9T01 cells (Table 1) that weakly express the NTM (Fig. 1) and ICN1 (Fig. 3). This variant has a rare missense change located in the epidermal growth factor-like repeat region of NEC, which was predicted not to affect protein function. It has been classified as a variant with uncertain significance (https://www.ncbi.nlm.nih.gov/clinvar/). Our data suggested that NOTCH1 p.Arg879Gln could be a loss-of-function mutation, and the entity described herein provides a model cell line for topics of clinical and scientific interest.

## Conclusions

Progression might be less dependent on the NOTCH1 signaling pathway in ATL cells than in T-ALL.

## Supplementary Information

**Additional file 1.**

**Additional file 2.**

**Additional file 3.**

**Additional file 4.**

**Additional file 5.**

## Data Availability

The datasets generated and analyzed during the current study are available in the DNA Data Bank of Japan repository at https://ddbj.nig.ac.jp/resource/biosample/SAMD00453566.

## Abbreviations

T-ALL: T-cell acute lymphoblastic leukemia/lymphoma
HD: Heterodimerization domain
ICN1: N-terminal intracellular domain 1
GSI: γ-secretase inhibitor
ATL: Adult T-cell leukemia/lymphoma
HTLV-1: T-cell leukemia virus type I
PBLs: Peripheral blood lymphocytes
Ab: Antibody
DAPT: N-[N-(3,5-difluorophenyl)-L-alanyl]-S-phenyl-glycine t-butyl ester
DMSO: Dimethyl sulfoxide
WES: Whole-exome sequencing
