# Supplementary figures and images for: Gamma-secretase inhibitor does not induce cytotoxicity in adult T-cell leukemia cell lines despite NOTCH1 expression
Source: BMC Cancer. 2022 Oct 15;22:1065. doi: 10.1186/s12885-022-10003-w (PMC9571424; doi:10.1186/s12885-022-10003-w)

Figure 1  
Full-length NOTCH1/NTM

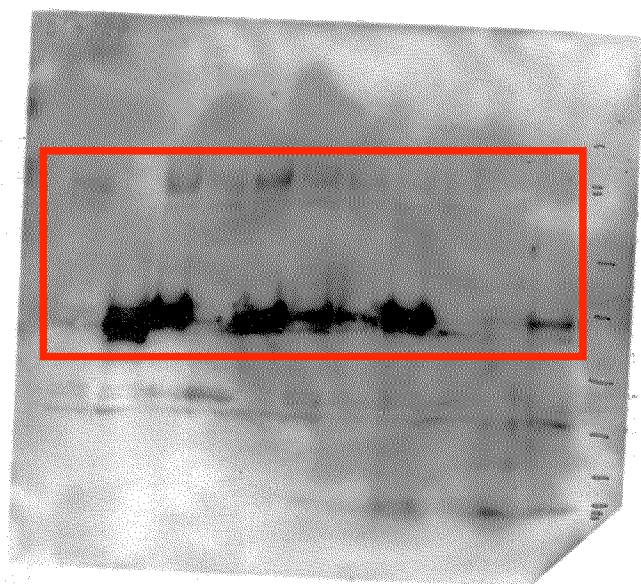

Notch1 24hrs. 060802

Supplement: Supplementary file 1 — Additional file 1. [file 12885_2022_10003_MOESM1_ESM.pdf]

Figure 3  
ICN1 (cleaved NOTCH1)  
Flip horizontal

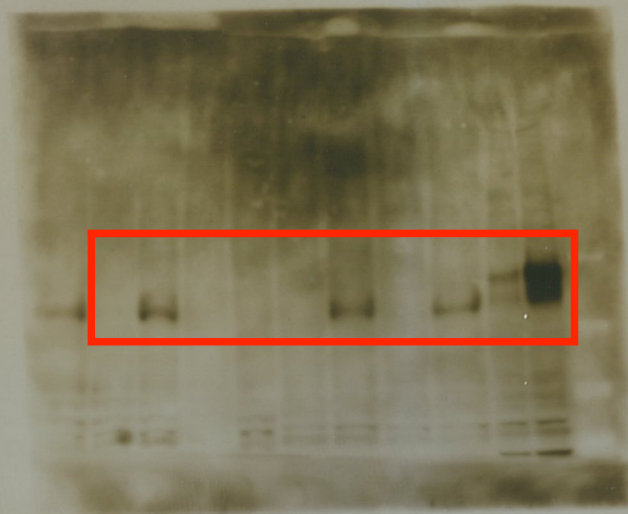

Supplement: Supplementary file 2 — Additional file 2. [file 12885_2022_10003_MOESM2_ESM.pdf]

Figure 1  
Actin

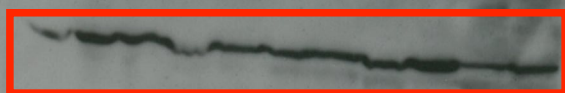

Actin

060819 20min

Supplement: Supplementary file 3 — Additional file 3. [file 12885_2022_10003_MOESM3_ESM.pdf]

Figure 2  
actin

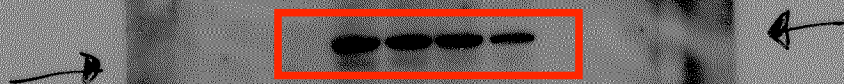

↑ ↑ ↑ ↑  
SIT neo 05 10 .

Actin-①  
060824 20min

Supplement: Supplementary file 4 — Additional file 4. [file 12885_2022_10003_MOESM4_ESM.pdf]

Figure 2  
full length NOTCH1/  
NTM

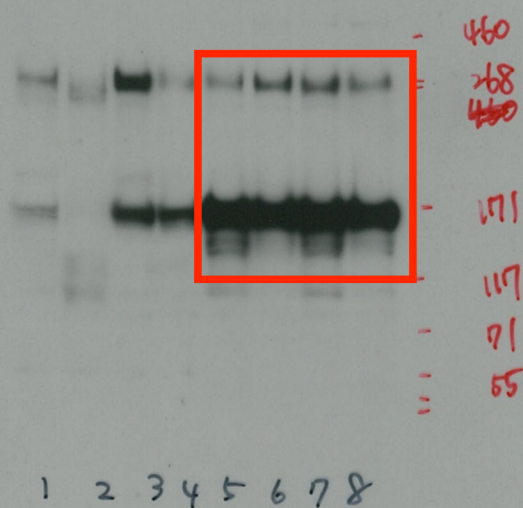

06/018 Notch1 ~~30~~ 3min.

Supplement: Supplementary file 5 — Additional file 5. [file 12885_2022_10003_MOESM5_ESM.pdf]
